# Multimorbidity in large Canadian urban centres: A multilevel analysis of pooled 2015–2018 cross-sectional cycles of the Canadian Community Health Survey

**DOI:** 10.1177/26335565211058037

**Published:** 2021-12-20

**Authors:** Piotr Wilk, Saverio Stranges, Rino Bellocco, Torsten Bohn, Hanen Samouda, Kathryn Nicholson, Tatjana T Makovski, Alana Maltby

**Affiliations:** 1 Department of Epidemiology and Biostatistics, Schulich School of Medicine and Dentistry, 6221 Western University, London, ON, Canada; 2 Department of Paediatrics, Schulich School of Medicine and Dentistry, Western University, London, ON, Canada; 3 Department of Family Medicine, Schulich School of Medicine and Dentistry, Western University, London, ON, Canada; 4 Department of Medical Epidemiology and Biostatistics, 27106 Karolinska Institute, Solna, Sweden; 5 Department of Population Health, Nutrition and Health Research Group, 58942 Luxembourg Institute of Health, Strassen, Luxembourg; 6 Department of Family Medicine, Care and Public Health Research Institute, 5211 Maastricht University, Maastricht, the Netherlands; 7 Chairgroup of Complex Genetics and Epidemiology, Nutrition and Metabolism in Translational Research, Care and Public Health Research Institute, Maastricht University, Maastricht, the Netherlands

**Keywords:** Multimorbidity, chronic conditions, prevalence, geographic variation, cross-sectional studies

## Abstract

**Background:**

There is limited knowledge on how the prevalence of multimorbidity varies within and across major Canadian urban centres. The objective of this study was to investigate the between-neighbourhood variation in the prevalence of multimorbidity in Canada’s large urban centres, controlling for compositional effects associated with individual-level demographic and socioeconomic factors.

**Methods:**

Cross-sectional data from the 2015–2018 cycles of the Canadian Community Health Survey (CCHS) were pooled at the microdata level. Respondents (20 years and older) residing in one of the 35 census metropolitan areas (CMAs) were included (*N* = 100,803). Census tracts (CTs) were used as a measure of neighbourhood. To assess the between-neighbourhood differences in multimorbidity prevalence, we fitted three sequential random intercept logistic regression models.

**Results:**

During the 2015–2018 period, 8.1% of residents of large urban centres had multimorbidity. The results from the unadjusted model indicate that 13.4% of the total individual variance in multimorbidity could be attributed to the between-neighbourhood differences. After adjustment for overall characteristics of the CMAs in which these neighbourhoods are located, as well as for individual-level demographic and socioeconomic factors related to compositional effects, 11.0% of the individual variance in multimorbidity could still be attributed to the between-neighbourhood differences.

**Conclusion:**

There is significant and substantial geographic variation in multimorbidity prevalence across neighbourhoods in Canada’s large urban centres. Residing in some neighbourhoods could be associated with increased odds of having multimorbidity, even after accounting for overall characteristics of the CMAs in which these neighbourhoods are located, as well as individual-level factors.

## Introduction

The number of Canadians living with chronic conditions poses a significant burden on the healthcare system, as well as on individuals and their families.^1–3^ A particular public health concern is the prevalence of multimorbidity, which is the co-existence of multiple chronic conditions within the same individual.^4–8^ The clustering of chronic conditions manifests differently across individuals and multimorbidity is likely to increase the complexity of disease prevention and clinical management due to the possibility of adverse health outcomes, frequent healthcare utilization, and greater healthcare needs.^7,9^ According to the Public Health Agency of Canada (PHAC),^10,11^ the prevalence of multimorbidity in Canada is increasing among individuals aged 20 years and older. Findings from the 2015 Canadian Community Health Survey (CCHS) showed that 6.9% of Canadian residents had at least two of the five following types of major chronic conditions: cardiovascular disease, cancer, chronic respiratory disease, diabetes, and mood and/or anxiety disorders. In addition, 15.8% had two or more of the following 10 common chronic conditions: heart disease, stroke, cancer, asthma, chronic obstructive pulmonary disease, diabetes, arthritis, Alzheimer’s disease or other dementia, mood disorders, and anxiety disorders.^11^ In 2017, these rates increased to 8.9% and 18.4%, respectively.^10^

Although health outcomes are related to the characteristics of the geographic areas in which people live,^12^ there is a dearth of knowledge on how the prevalence of multimorbidity varies within and across major Canadian urban centres. Few studies have explored geographic variation in multimorbidity prevalence in Canada, focusing on specific provinces such as Ontario or British Columbia.^13,14^ Over the past century, the proportion of Canadians living in urban settings has steadily increased, mostly as a result of economic opportunities.^15^ In 2016, 70.4% (24,945,123) of Canadians lived in one of the 35 census metropolitan areas (CMAs).^16^ Census metropolitan areas are defined as large urban areas with a total population of at least 100,000, with a minimum of 50,000 residing in a population centre (also known as the core).^17^

The geographic variation in multimorbidity prevalence may be linked to compositional effects related to self-selection of individuals with specific characteristics that reside in a specific neighbourhood and CMA.^18^ For example, the between-neighbourhood variation in multimorbidity prevalence could be partially due to disproportional concentrations of individuals with similar demographic or socioeconomic characteristics in a specific neighbourhood.

The overall prevalence of multimorbidity may not adequately reflect the burden of this condition at the local level;^19^ therefore, further research is needed to better understand how multimorbidity prevalence varies within and across large urban centres (CMAs) in Canada. There have been no national studies examining the between-neighbourhood differences in multimorbidity and whether these differences can be accounted by compositional effects. Thus, the objective of this study was to investigate geographic variation in the prevalence of multimorbidity in Canada’s large urban centres, focusing on the between-neighbourhood differences. We also assessed if this variation can be accounted by individual-level demographic and socioeconomic factors associated with compositional effects.

## Methods

For this study, we used cross-sectional survey data that is routinely collected in Canada. The study is reported according to the REporting of studies Conducted using Observational Routinely-collected health Data (RECORD) guidelines.^20^

### Data and study population

The CCHS is the largest national cross-sectional survey in Canada that collects information on health status, health system utilization, and health determinants since 2001.^21^ The CCHS data are primarily used for health surveillance and population health research. The survey is representative of individuals 12 years and older living within the 10 provinces and three territories of Canada. Data are collected using computer assisted personal interviewing software and telephone interviews. A participant may be included in more than one related survey; however, this could not be assessed (more details on the survey methodology can be found online).^21^ Data from respondents 20 years and older residing in one of the 35 CMAs were included in the current study. To increase the sample size of respondents from each neighbourhood, data from the four independent annual cycles of the CCHS, 2015, 2016, 2017 and 2018, were pooled at the micro-data level using the methods proposed by Thomas and Wannell.^22^ These four cycles used an identical sampling design and consistent population representation. The obtained estimates are time period estimates and should be interpreted as attributes of the average population residing in the 35 CMA during the 2015–2018 time period.^22^

### Multimorbidity

Consistent with the public health definition of multimorbidity adopted in Canada,^10^ CCHS respondents were asked about specific long-term conditions that were ‘expected to last or have already lasted 6 months or more and that have been diagnosed by a health professional.’ Based on the classification system used by PHAC^10^ and implemented in the CCHS questionnaire, multimorbidity was defined as the co-occurrence of a least two of the five groups of chronic conditions: cancer (ever had), diabetes, cardiovascular disease (heart disease and/or stroke), chronic respiratory disease (asthma and/or chronic obstructive pulmonary disease), and mental illnesses (mood and/or anxiety disorders). Respondents with missing values for multimorbidity (*N* = 16) were excluded from the analysis.

### Neighbourhoods

For this study, Census tracts (CTs) were used as a measure of neighbourhood in each of 35 CMAs. Census tracts are relatively small and stable geographic areas nested within CMAs with population counts of less than 10,000 persons. Census tracts are commonly used as a proxy measure for neighbourhood.^19,23^ The boundaries of CTs are delineated by Statistics Canada in partnership with local community stakeholders (i.e. planners, health and social workers, and educators). Census tracts follow permanent and easily recognizable physical features and these boundaries are rarely revised in order to maintain data comparability between censuses.^23^

### Individual-level confounders

Several relevant demographic and socioeconomic factors (age, sex, ethno-cultural background, immigration status, education and household income), previously identified in the literature,^24–27^ have been investigated as potential individual-level confounders and were included in this study to control for compositional effects. Age was operationalized as an ordinal categorical variable (20–44, 45–64, 65–84, and 85 and over). Ethno-cultural background was assessed by a nominal categorical variable: white, black, East Asian/South-east Asian, Aboriginal and others. Immigration status was operationalized as a nominal categorical variable: born in Canada, established immigrants (5 or more years in Canada) and recent immigrants (less than 5 years in Canada).^26^ Education was assessed as an ordinal categorical variable: lower than secondary school diploma, secondary school completion and any post-secondary education. Relative gross household income was categorized in quintiles: lowest quintile, low-middle quintile, middle quintile, high-middle quintile and highest quintile. Three of these confounders had some missing data related to item non-response: ethno-cultural background (3.02%), immigration status (2.53%) and education (1.64%) We created separate categories for these three variables to represent non-respondents.

### Statistical analysis

Frequency distributions for categorical variables and descriptive statistics (means and standard deviations [SD]) for continuous variables were computed to describe the CCHS sample (Table 1). To address the study objective (i.e. assessing the between-neighbourhood differences in multimorbidity prevalence), we fitted three sequential random intercept multi-level logistic regression models. We first ran the unadjusted model (Model 1) to estimate the overall level of between-neighbourhood variation in multimorbidity prevalence. In this model, we controlled only for factors related to the survey design (i.e. the survey year [2015, 2016, 2017 and 2018], the interview mode [in person vs. by telephone], and the interview type [by proxy vs. with respondent]).^22^ In the second model (Model 2), we added a series of dummy (binary) variables representing 34 CMAs in Canada (Toronto, Canada’s largest CMA, was used as the reference group). The objective of this model was to control for the overall effect of residence in each of the 35 CMAs and to assess if the unadjusted between-neighbourhood differences in multimorbidity prevalence estimated in Model 1 might be accounted for by the overall characteristics of CMAs. In the third model (Model 3), we added several individual-level demographic and socioeconomic confounders (age, sex, ethno-cultural background, immigration status, education and household income). The results from this adjusted model allowed us to assess if the observed between-neighbourhood differences in multimorbidity prevalence could be explained by the compositional effects. We assumed that these individual-level confounders had fixed effects on the outcome variable (i.e. their effects did not differ across neighbourhoods).

**Table 1.** Descriptive statistics.

Individual-level factors	Total population	
	Frequency (*N*)	Percent (%)
Multimorbidity		
Yes	8125	8.1
No	92,678	92.0
Non-respondents	16	
Survey year		
2015	23,627	23.4
2016	25,814	25.6
2017	26,491	26.3
2018	24,887	24.7
Interview type		
Respondent	78,477	77.8
Proxy	22,342	22.2
Interview mode		
By telephone	97,898	97.1
In person	2921	2.9
Age		
20–44 years	45,900	45.5
45–64 years	35,397	35.1
65–84 years	17,663	17.5
85 + years	1859	1.8
Sex		
Female	51,451	51.0
Male	49,368	48.9
Ethno-cultural background		
White	67,492	66.9
Black	3358	3.3
East Asian/South-East Asian	10,015	9.9
Aboriginal	2617	2.6
Other^a^	14,292	14.2
Non-respondents	3046	3.0
Immigration status		
Non-immigrant	84,646	84.0
≥5 years in Canada	12,867	12.8
<5 years in Canada	754	0.8
Non-respondents	2552	2.5
Education		
Less than secondary school	9497	9.4
Secondary school	21,551	21.4
Post-secondary	68,121	67.6
Non-respondents	1650	1.6
Household income		
Lowest quintile	19,805	19.6
Low-middle quintile	20,043	19.9
Middle quintile	20,444	20.3
High-middle quintile	20,441	20.3
Highest quintile	20,087	19.9

^a^ ‘Other’ category in Ethno-cultural Background consists of all other groups with small frequency counts (e.g. Chinese, Korean, Filipino, Japanese, Arab, West Asian, Latin American).

For multilevel logistic regression analysis, there is no agreement on the reporting of results when assessing general and specific contextual effects. Therefore, we followed the recommendations from Merlo and colleagues.^28^ To quantify the magnitude of the between-neighbourhood variation in multimorbidity (i.e. general neighbourhood effect), we reported (1) the variance of the random intercept (τ^2^) with its standard error (SE); (2) the variance partition coefficient (VPC), calculated using the latent variable method for rescaling individual-level variances;^29^ and (3) the median odds ratios (MOR),^30^ which represent the between-neighbourhood variances on an odds ratio scale. The key advantage of the MOR is that it is directly comparable to the odds ratios for individual-level fixed effects. Model results are presented in Table 2 (random effects and fit statistics) and Table 3 (fixed effects).

**Table 2.** Random effects and fit statistics.

Model	Model 1	Model 2	Model 3
Variance	0.506	0.465	0.404
SE	0.027	0.025	0.024
Z-Value	18.810	18.370	16.880
Pr > Z	<0.0001	<0.0001	<0.0001
VPC	0.134	0.124	0.110
MOR	1.971	1.916	1.833
Pearson chi-square	87,399	87,773	83,916
Pearson chi-square/DF	0.87	0.87	0.83

Legend: SE: standard error; VPC: variance partition coefficient; MOR: median odds ratio; DF: degrees of freedom.

**Table 3.** Fixed effects (Model 3 only).

Variable	Log odds	SE	Odds [95% CI]
*Intercept*	−3.714	0.065	
Survey Year			
2015	0.113	0.039	1.120 [1.038–1.208]
2016	0.026	0.036	1.026 [0.956–1.101]
2017	−0.018	0.036	0.982 [0.916–1.053]
2018	0.000		
Interview Mode			
In person	0.049	0.033	1.050 [0.985–1.119]
By telephone (REF)	0.000		
Interview Type			
Proxy	0.954	0.053	2.596 [2.338–2.882]
Respondent (REF)	0.000		
Census metropolitan area			
St. John’s	0.018	0.177	1.018 [0.720–1.441]
Halifax	0.233	0.128	1.262 [0.983–1.621]
Moncton	0.329	0.207	1.389 [0.925–2.085]
Saint John	0.341	0.197	1.407 [0.956–2.070]
Saguenay	0.051	0.184	1.052 [0.734–1.509]
Québec City	−0.280	0.111	0.756 [0.608–0.939]
Sherbrooke	−0.302	0.182	0.739 [0.518–1.055]
Trois-Rivières	−0.147	0.204	0.863 [0.578–1.289]
Montréal	−0.165	0.064	0.848 [0.748–0.961]
Ottawa/Gatineau	0.095	0.088	1.099 [0.926–1.306]
Kingston	0.301	0.185	1.351 [0.941–1.941]
Belleville	0.408	0.220	1.503 [0.977–2.311]
Peterborough	0.472	0.213	1.603 [1.055–2.435]
Oshawa	0.305	0.129	1.356 [1.053–1.746]
Hamilton	0.097	0.108	1.102 [0.892–1.360]
St. Catharines/Niagara	0.134	0.128	1.144 [0.890–1.469]
Kitchener/Cambridge/Waterloo	0.334	0.120	1.396 [1.104–1.765]
Brantford	0.290	0.208	1.337 [0.889–2.011]
Guelph	0.162	0.223	1.176 [0.760–1.820]
London	0.216	0.118	1.241 [0.985–1.564]
Windsor	0.197	0.142	1.217 [0.922–1.607]
Barrie	0.604	0.191	1.830 [1.258–2.663]
Greater Sudbury	0.397	0.170	1.488 [1.066–2.076]
Thunder Bay	0.074	0.206	1.077 [0.719–1.613]
Winnipeg	−0.015	0.102	0.985 [0.807–1.203]
Regina	0.192	0.168	1.212 [0.873–1.683]
Saskatoon	−0.153	0.167	0.858 [0.619–1.190]
Lethbridge	−0.136	0.243	0.873 [0.542–1.406]
Calgary	−0.061	0.092	0.941 [0.785–1.127]
Edmonton	0.190	0.089	1.209 [1.015–1.440]
Kelowna	−0.053	0.198	0.948 [0.644–1.397]
Abbotsford/Mission	0.323	0.187	1.382 [0.957–1.994]
Vancouver	0.055	0.073	1.056 [0.916–1.218]
Victoria	−0.017	0.142	0.983 [0.744–1.298]
Toronto (REF)	0.000		
Age			
20–44 years (REF)	0.000		
45–64 years	1.039	0.034	2.825 [2.642–3.021]
65–84 years	1.683	0.036	5.379 [5.009–5.776]
85 + years	1.699	0.069	5.467 [4.773–6.262]
Sex			
Female	0.179	0.025	1.197 [1.138–1.258]
Male (REF)	0.000		
Ethno-cultural Background			
White (REF)	0.000		
Black	−0.481	0.090	0.618 [0.518–0.738]
East Asian/South-east Asian	−0.832	0.063	0.435 [0.385–0.492]
Aboriginal	0.603	0.064	1.828 [1.613–2.073]
Other	−0.452	0.048	0.636 [0.578–0.699]
Non-respondents	−0.239	0.148	0.788 [0.589–1.053]
Immigration Status			
Non-immigrant (REF)	0.000		
≥5 years in Canada	−0.438	0.053	0.645 [0.582–0.716]
<5 years in Canada	−1.254	0.307	0.285 [0.156–0.521]
Non-respondents	−0.180	0.160	0.835 [0.611–1.143]
Education			
Less than secondary school	0.373	0.037	1.451 [1.350–1.561]
Secondary school	0.140	0.031	1.150 [1.082–1.222]
Post-secondary	0.000		
Non-respondents	0.221	0.086	1.247 [1.054–1.475]
Household Income			
Lowest quintile	0.604	0.038	1.830 [1.698–1.973]
Low-middle quintile	0.186	0.039	1.204 [1.115–1.300]
Middle quintile (REF)	0.000		
High-middle quintile	−0.212	0.043	0.809 [0.744–0.879]
Highest quintile	−0.411	0.045	0.663 [0.607–0.725]

Legend: SE: standard error; CI: confidence interval; REF: reference category.

Sampling weights and bootstrap weights were used to extrapolate the results to the individuals residing in Canada’s CMAs during the 2015–2018 time period and to account for the CCHS sampling design. Analysis was conducted in SAS 9.4 and PROC GLIMMIX procedures were used.^31^

## Results

### Descriptive statistics

The sample included in this analysis consisted of 100,803 respondents. Table 1 reports the descriptive statistics. Approximately half of the sample consisted of women (51.0%), and the average age was 47.86 years. As presented in Table 1, 8.1% (95% CI = 7.82, 8.30) of the residents of large urban centres in Canada reported having multimorbidity. This prevalence, however, is higher among women (8.9%; 95% CI = 8.60–9.30) than among men (7.1%; 95% CI = 6.75–7.41).

### Between-neighbourhood differences in multimorbidity

The fit statistics reported in Table 2, which take into account the random effects, suggest that all three multilevel models fit the data well and that there is no residual overdispersion; the ratios of the Pearson chi-square statistics to their degrees of freedom are 0.87, 0.87 and 0.83, respectively. In the unadjusted multilevel logistic model, which included only the survey design variables (Model 1), the between-neighbourhood variance in the outcome variable of 0.506 (SE = 0.027) suggested a statistically significant overall between-neighbourhood variance in the odds of having multimorbidity (see Table 2). The VPC for this estimate indicates that 13.4% of the variation in the outcome variable can be attributed to the between-neighbourhood differences. The MOR of 1.971 implies that if a randomly selected resident moves from a neighbourhood with a lower likelihood of multimorbidity to a neighbourhood with a higher likelihood, their odds of having multimorbidity would be almost doubled. The results from Model 2 suggest that the inclusion of dummy variables for the CMAs (i.e. to account for overall CMA characteristics) reduced the size of the between-neighbourhood variance from 0.506 to 0.465 (SE = 0.025). The residual VPC for Model 2 suggests that the overall CMA characteristics account only for a small portion of the between-neighbourhood variance in multimorbidity and that, when these characteristics are accounted for, 12.4% of the variance in the outcome variable can still be attributed to the between-neighbourhood differences. The MOR for the between-neighbourhood variance in Model 2 was 1.916. In Model 3, the inclusion of individual-level confounders (i.e. sex, age, ethno-cultural background, immigration status, education and household income) to account for potential compositional effects further attenuated the size of the between-neighbourhood variance from 0.464 to 0.404 (SE = 0.024). The residual VPC for Model 3 suggests that, after accounting for these compositional effects, 11.0% of the variance in the outcome variable can still be attributed to the general neighbourhood effect; the MOR for this between-neighbourhood variance was 1.833.

### Differences in multimorbidity prevalence across CMAs

**Figure 1.**
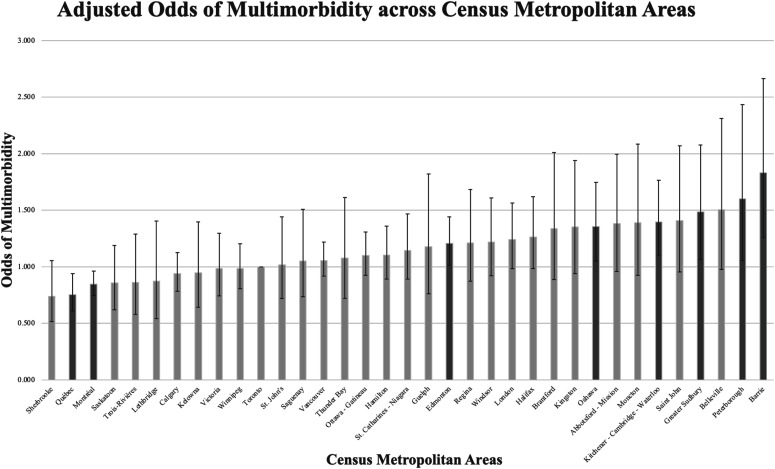
Adjusted odds of multimorbidity across census metropolitan areas (2015–2018) - Error bars show 95% confidence intervals.

The results from Model 3 suggest that there were significant differences in the adjusted odds (Figure 1) of multimorbidity across the 35 CMAs, controlling for the neighbourhood of residence and the individual-level confounders. Compared to the residents of Toronto, individuals residing in two CMAs in the province of Québec, Québec City (OR = 0.756; 95% CI = 0.608, 0.939) and Montréal (OR = 0.848; 95% CI = 0.748, 0.961) had lower odds of multimorbidity. In addition, five CMAs in the province of Ontario, Oshawa (OR = 1.356; 95% CI = 1.053, 1.746), Kitchener-Cambridge-Waterloo (OR = 1.396; 95% CI = 1.104, 1.765), Greater Sudbury (OR = 1.488; 95% CI = 1.066, 2.076), Peterborough (OR = 1.603; 95% CI = 1.055, 2.435) and Barrie (OR = 1.830; 95% CI = 1.258, 2.663), as well as one CMA in the province of Alberta, Edmonton (OR = 1.209; 95% CI = 1.015, 1.440), had higher odds of multimorbidity than Toronto.

### Individual-level demographic and socioeconomic confounders

The results from Model 3 can also be used to assess the relationship between individual-level demographic and socioeconomic factors and the odds of having multimorbidity. These results suggest that, compared to 20–44-year-old urban dwellers, the odds of having multimorbidity were higher among individuals of 45–64 (OR = 2.825; 95% CI = 2.642, 3.021), 65–84 (OR = 5.379; 95% CI = 5.009, 5.776) and 85 or more years of age (OR = 5.467; 95% CI = 4.773, 6.262). The ORs for age were greater than the MORs, which indicates that the unexplained between-neighbourhood variance was less relevant than this individual-level factor for understanding rates of multimorbidity. Compared to men, women were 19.7% more likely to have multimorbidity (OR = 1.197; 95% CI = 1.138, 1.258). Recent immigrants were 71.5% (OR = 0.285; 95% CI = 0.156, 0.521) and established immigrants 35.5% (OR = 0.645; 95% CI = 0.582, 0.716) less likely to have multimorbidity than individuals born in Canada. Compared with urban dwellers with post-secondary education, individuals with less than secondary education and individuals with secondary school diploma were 45.1% and 15.0% more likely, respectively, to have multimorbidity. Compared to the respondents who identified themselves as ‘white’, individuals who identified themselves as Aboriginal were 82.8% more likely to have multimorbidity. The members of some ethno-cultural groups reported lower odds of multimorbidity than ‘white’ respondents: black respondents by 38.2%, East Asian/South-east Asian by 56.5%, and members of other ethno-cultural groups by 36.4%. Compared to the individuals in the middle household income quintile, residents of large urban areas in the lowest quintile were 83.0% more likely to have multimorbidity (OR = 1.830; 95% CI = 1.698, 1.973). In comparison, individuals in the highest income quintile were 33.7% less likely to have multimorbidity (OR = 0.663; 95% CI = 0.607, 0.725).

## Discussion

The overall objective of the current study was to assess the between-neighbourhood differences in the prevalence of multimorbidity in Canada’s large urban centres, controlling for compositional effects associated with individual-level demographic and socioeconomic factors. The key finding of this study is that there is statistically significant and substantial geographic variation in the prevalence of multimorbidity across neighbourhoods located in large urban centres. Specifically, the results from Model 1 indicate that, without adjusting for the overall characteristics of CMAs in which these neighbourhoods are located and for individual-level confounders related to compositional effects, as much as 13.4% of the variation in multimorbidity prevalence may be attributed to the between-neighbourhood differences (i.e. general compositional effect). Upon considering the overall characteristics of CMAs in Model 2, the between-neighbourhood variance decreased slightly to 12.4%; that is, the overall characteristics of the CMAs did not explain a substantial portion of the between-neighbourhood variance. As this variance could be due to the differences in the distribution of individual-level characteristics across neighbourhoods, several individual-level confounders related to the compositional effects (i.e. age, sex, ethno-cultural background, immigration status, education and household income) were adjusted for in Model 3. After these factors were accounted for, the between-neighbourhood variance was further reduced by 1.4%, although it is possible that the remaining variance is still related to differences in other individual-level characteristics not considered in this study, such as health-related behaviours (e.g. nutritional status, physical activity, smoking, sleep patterns, etc.) and/or health care access.

It could be hypothesized that these between-neighbourhood differences in multimorbidity prevalence might at least be partially explained by neighbourhood-level social (e.g. deprivation, social interactions), built (e.g. urban form) and natural (e.g. pollution, noise, or climate) environmental characteristics. For example, in two studies from Ontario,^13,24^ the prevalence of multimorbidity was higher in areas with the greatest level of deprivation compared to the areas that were the least deprived. Moin and colleagues found that in the most deprived areas, cases of multimorbidity were occurring nearly 10 years sooner compared to areas with the least deprivation.^24^ As neighbourhoods provide the availability of and access to resources that ultimately shape the health and well-being of the individuals and families living in these geographically defined areas,^32,33^ the geographic patterning of health outcomes and inequalities may reflect the variation in these resources.^32^ However, the relationship between neighbourhood-level factors and multimorbidity is not as well investigated as the impact of individual characteristics. By developing a better understanding of variation in multimorbidity using policy relevant units of geography (i.e. neighbourhoods and CMAs), the current findings may be used to inform local public health officials and decision makers about the burden of chronic conditions and inequalities in multimorbidity and subsequently enable them to better organize the allocation of resources and to develop more targeted interventions.

Another key finding of this study is that there were substantial differences in multimorbidity across CMAs. In the adjusted models, controlling for the general neighbourhood effects and individual-level confounders, the odds of multimorbidity were 1.83 times greater in Barrie, Ontario, the CMA with highest odds of multimorbidity, than in Toronto (the largest CMA). Edmonton, Greater Sudbury, Kitchener-Cambridge-Waterloo, Oshawa and Peterborough also had greater odds of multimorbidity than Toronto, whereas residents in Québec City and Montréal had lower odds of multimorbidity than Toronto. Previous research assessing the differences in health and health behaviours across CMAs has indicated that there is a significant variation in self-reported health, adoption of healthy behaviours, perception of life stress and smoking.^34^ Canada’s universal health coverage guarantees reasonable access to medically necessary health services. However, provinces and territories finance, regulate, and administer healthcare coverage to their residents and may differ in how they allocate healthcare resources.^35^ Differences in health across CMAs may be a result of several other factors including, but not limited to, the demographics and socioeconomic characteristics of the area, or the access to health care.^12,33^

Previous literature has indicated that the likelihood of multimorbidity is greater among certain demographic and socioeconomic groups. In the current study, the ORs for age were greater than the MORs, indicating that age may be more relevant for understanding geographic variation in rates of multimorbidity than characteristics of the geographic areas. When controlling for other individual-level factors, the odds of multimorbidity increased with age, which is a well-established determinant.^9,26,36,37^ Being female also increased the likelihood of having multimorbidity. In a systematic review, Violan et al.^36^ indicated that after adjusting for age and gender, studies showed an increased prevalence of multimorbidity among women and this could be explained by their greater healthcare utilization which may result in higher rates of diagnosis, the inclusion of gender-specific conditions in the study, or actual differences in the burden of chronic conditions. Further examination of this relationship is warranted. Similar to the findings of Roberts et al.^26^ and Moin et al.,^24^ recent and established immigrants were found to be less likely to have multimorbidity than those who were born in Canada. This finding may be evidence of the healthy immigrant effect, which suggests that immigrants’ health is better than that of residents born in Canada, although it tends to decline after increased time living in Canada.^38^ Prior to entering Canada, immigrants are subject to strict screening processes, including a medical assessment, which ensures they do not pose a risk to public health or cause increased demand on health and social services.^39^ Ahmed et al. noted that barriers to access to primary health care in Canada related to communication, socio-economic status and immigrant knowledge of the healthcare system may in turn result in multimorbidity being underdiagnosed among this population.^40^ Certain ethno-cultural groups (i.e. black and East Asian/South-east Asian) had lower odds of multimorbidity compared to individuals who identified as ‘white’. However, individuals who identified as Aboriginal were more likely (82.8%) to report multiple chronic conditions than individuals who identified as ‘white’, and this finding has been echoed in other Canadian research.^26^ Consistently, there has been an inverse relationship between socioeconomic factors and multimorbidity,^27,36^ as the onset of multimorbidity occurs earlier among individuals in more socially deprived groups.^26^ In our study, having less than secondary education increased the odds of having multimorbidity. Also, there was a clear gradient in the effect of household income, as the odds of multimorbidity decreased with each income quintile. The study participants in the lowest quintile of household income were 83.0% more likely to have multimorbidity compared to the individuals in the middle-income quintile. In an Ontario study of the determinants of inequality in multimorbidity, using decomposition analyses, Mondor et al. found that household income accounted for nearly 70% of the inequality in multimorbidity occurrence.^25^

### Strengths and Limitations

To our knowledge, this is the first Canadian study to assess the geographic variation in multimorbidity prevalence across neighbourhoods in large urban centres. With Canada becoming increasingly urban, focusing on the urban environment where there is more variation in health status than across provincial boundaries is needed to better understand and reduce health inequalities and multimorbidity.^12,19^ However, this study is not without limitations. First, the CCHS is a cross-sectional survey, and therefore, we cannot infer causation. Additionally, the CCHS contains self-reported data on diagnosed chronic conditions and might be subject to multiple biases, such as recall or social desirability bias. Moreover, there are a limited number of chronic conditions included in the CCHS. In our study, multimorbidity was measured based on a commonly used definition for population-based surveys,^10^ to align with other Canadian studies using the same dataset. However, using different measurements of multimorbidity such as those more commonly used in primary care settings or administrative databases (i.e. including a greater number of chronic conditions to assess multimorbidity) might elicit different results.^41,42^ Lastly, we used CTs as the geographic unit of analysis and a proxy for neighbourhoods. Although we believe it was a reasonable decision as previous research has indicated CTs are appropriate proxies for natural neighbourhood boundaries,^19^ using other geographic units could have generated different results.

In conclusion, there is a significant and substantial geographic variation in multimorbidity prevalence across neighbourhoods in Canada’s large urban centres. The findings from this study suggest that residence in some neighbourhoods is associated with higher odds of multimorbidity. Although the findings are specific to Canada, this paper contributes to the limited research available on geographic variation in multimorbidity and the methods employed can be replicated in other jurisdictions. To develop a bettering understanding of why some geographic areas have a higher prevalence of multimorbidity, there is a need to explore the role of neighbourhood contextual factors as they are associated with health inequalities, particularly in urban settings. Additionally, targeted analysis focused on geographic differences in specific chronic conditions should also be conducted.

## References

[1] Broemeling A-M Watson DE Prebtani F . Population patterns of chronic health conditions, co-morbidity and healthcare use in Canada: implications for policy and practice. Healthc Q 2008; 11: 70–76. DOI: 10.12927/hcq.2008.19859. 18536538

[2] Terner M Reason B McKeag AM , et al. Chronic conditions more than age drive health system use in Canadian seniors. Healthc Q 2011; 14: 19–22. DOI: 10.12927/hcq.2011.22485. 21841372

[3] Zhang W Sun H . Formal and informal care received by middle-aged and older adults with chronic conditions in Canada: CLSA data. PLoS One 2020; 15: e0235774. DOI: 10.1371/journal.pone.0235774. 32634161 PMC7340302

[4] Mercer S Salisbury C Fortin M . ABC of multimorbidity. Oxford, UK: John Wiley & Sons, 2014.

[5] Moffat K Mercer SW . Challenges of managing people with multimorbidity in today’s healthcare systems. BMC Fam Pract 2015; 16: 129. DOI: 10.1186/s12875-015-0344-4. 26462820 PMC4604728

[6] van den Akker M Buntinx F Knottnerus JA . Comorbidity or multimorbidity. Eur J Gen Pract 2009; 2: 65–70. DOI: 10.3109/13814789609162146.

[7] Boyd CM Fortin M . Future of multimorbidity research: how should understanding of multimorbidity inform health system design? Public Health Rev 2010; 32: 451–474.

[8] Nicholson K Makovski TT Griffith LE , et al. Multimorbidity and comorbidity revisited: refining the concepts for international health research. J Clin Epidemiol 2018; 105: 142–146. DOI: 10.1016/j.jclinepi.2018.09.008. 30253215

[9] Koné Pefoyo AJ Bronskill SE Gruneir A , et al. The increasing burden and complexity of multimorbidity. BMC Public Health 2015; 15. DOI: 10.1186/s12889-015-1733-2. PMC4415224 25903064

[10] Public Health Agency of Canada . Canadian Chronic Disease Indicators, Quick Stats, 2019 Edition, https://www.canada.ca/content/dam/phac-aspc/documents/services/reports-publications/health-promotion-chronic-disease-prevention-canada-research-policy-practice/vol-39-no-10-2019/EN_2_Varin.pdf (2019, accessed 10 November 2020).

[11] Public Health Agency of Canada . Canadian Chronic Disease Indicators, Quick Stats, 2017 Edition. https://www.canada.ca/content/dam/phac-aspc/documents/services/publications/health-promotion-chronic-disease-prevention-canada-research-policy-practice/vol-37-no-8-2017/ar-03-eng.pdf (2017, accessed 10 November 2020).

[12] Canadian Institute for Health Information . Reducing Gaps in Health: A Focus on Socio-Economic Status in Urban Canada. https://secure.cihi.ca/free_products/Reducing_Gaps_in_Health_Report_EN_081009.pdf (2008, accessed 12 November 2020).

[13] Ryan BL Bray Jenkyn K Shariff SZ , et al. Beyond the grey tsunami: a cross-sectional population-based study of multimorbidity in Ontario. Can J Public Health 2018; 109: 845–854. DOI: 10.17269/s41997-018-0103-0. 30022403 PMC6964436

[14] Basham CA . Regional variation in multimorbidity prevalence in british columbia, canada: a cross-sectional analysis of canadian community health survey data, 2015/16. Health Promot Chronic Dis Prev Can 2020; 40: 225–234. DOI: 10.24095/hpcdp.40.7/8.02. 32667879 PMC7450904

[15] Statistics Canada . Canada goes urban. https://www150.statcan.gc.ca/n1/pub/11-630-x/11-630-x2015004-eng.htm (2018, accessed 25 September 2018).

[16] Statistics Canada . Annual demographic estimates: subprovincial areas, July 1, 2016 - section 1: census metropolitan areas, https://www150.statcan.gc.ca/n1/pub/91-214-x/2017000/section01-eng.htm (2017, accessed 10 February 2020).

[17] Statistics Canada . Dictionary, census of population, 2016 - census metropolitan area (CMA) and census agglomeration (CA). https://www12.statcan.gc.ca/census-recensement/2016/ref/dict/geo009-eng.cfm (2016, accessed 10 November 2020).

[18] Congdon P . A multilevel model for comorbid outcomes: obesity and diabetes in the US. Int J Environ Res Public Health 2010; 7: 333–352. DOI: 10.3390/ijerph7020333. 20616977 PMC2872282

[19] Ross NA Tremblay SS Graham K . Neighbourhood influences on health in Montreal, Canada. Social Sci Med 2004; 59: 1485–1494. DOI: 10.1016/j.socscimed.2004.01.016. 15246176

[20] Benchimol EI Smeeth L Guttmann A , et al. The reporting of studies conducted using observational routinely-collected health data (RECORD) statement. PLOS Med 2015; 12: e1001885. DOI: 10.1371/journal.pmed.1001885. 26440803 PMC4595218

[21] Statistics Canada . Canadian community health survey - annual component (CCHS). https://www23.statcan.gc.ca/imdb/p2SV.pl?Function=getSurvey&Id=1263799 (2020, accessed 28 August 2020).

[22] Thomas S Wannell B . Combining cycles of the Canadian community health survey. Health Rep 2009; 20: 53–58. 19388369

[23] Statistics Canada . Dictionary, Census of Population, 2016 - Census tract (CT), 2016, https://www12.statcan.gc.ca/census-recensement/2016/ref/dict/geo013-eng.cfm (2016, accessed 10 November 2020).

[24] Moin JS Moineddin R Upshur REG . Measuring the association between marginalization and multimorbidity in Ontario, Canada: a cross-sectional study. J Comorbidity 2018; 8: 2235042X18814939. DOI: 10.1177/2235042X18814939. PMC6295698 30574456

[25] Mondor L Cohen D Khan AI , et al. Income inequalities in multimorbidity prevalence in Ontario, Canada: a decomposition analysis of linked survey and health administrative data. Int J Equity Health 2018; 17. DOI: 10.1186/s12939-018-0800-6. PMC6019796 29941034

[26] Roberts KC Rao DP Bennett TL , et al. Prevalence and patterns of chronic disease multimorbidity and associated determinants in Canada. Health Promot Chronic Dis Prev Can 2015; 35: 87–94. 26302227 10.24095/hpcdp.35.6.01 PMC4910465

[27] Pathirana TI Jackson CA . Socioeconomic status and multimorbidity: a systematic review and meta-analysis. Aust New Zealand J Public Health 2018; 42: 186–194. DOI: 10.1111/1753-6405.12762. 29442409

[28] Merlo J Viciana-Fernandez FJ Ramiro-Farinas D , et al. Bringing the individual back to small-area variation studies: a multilevel analysis of all-cause mortality in Andalusia, Spain. Social Sci Med 2012; 75: 1477–1487. DOI: 10.1016/j.socscimed.2012.06.004. 22795359

[29] Snijders T Bosker R . Statistical treatment of clustered dataMultilevel analysis—an introduction to basic and advanced multilevel modeling. Thousand Oaks, CA: Sage, 1999, p. 13–37.

[30] Merlo J Chaix B Ohlsson H , et al. A brief conceptual tutorial of multilevel analysis in social epidemiology: using measures of clustering in multilevel logistic regression to investigate contextual phenomena. J Epidemiol Community Health 2006; 60: 290–297. DOI: 10.1136/jech.2004.029454. 16537344 PMC2566165

[31] SAS Institute Inc . SAS 9.4. Cary, NC: SAS Institute Inc., 2016.

[32] Bernard P Charafeddine R Frohlich KL , et al. Health inequalities and place: a theoretical conception of neighbourhood. Social Sci Med 2007; 65: 1839–1852. DOI: 10.1016/j.socscimed.2007.05.037. 17614174

[33] Shah TI Bell S Wilson K . Spatial accessibility to health care services: identifying under-serviced neighbourhoods in Canadian urban areas. PLoS One 2016; 11: e0168208. DOI: 10.1371/journal.pone.0168208. 27997577 PMC5172578

[34] Canadian Institute for Health Information . Improving the health of Canadians: an introduction to health in urban places. https://secure.cihi.ca/free_products/PH_Full_Report_English.pdf (2006, accessed 08 December 2020).

[35] Marchildon GP Allin S Merkur S . Health system review. Health Syst Transit 2020; 22: 1–194. 33527903

[36] Violan C Foguet-Boreu Q Flores-Mateo G , et al. Prevalence, determinants and patterns of multimorbidity in primary care: a systematic review of observational studies. PLoS One 2014; 9: e102149. DOI: 10.1371/journal.pone.0102149. 25048354 PMC4105594

[37] Feely A Lix LM Reimer K . Estimating multimorbidity prevalence with the Canadian chronic disease surveillance system. Health Promot Chronic Dis Prev Can 2017; 37: 215–222. DOI: 10.24095/hpcdp.37.7.02. 28703703 PMC5650032

[38] McDonald JT Kennedy S . Insights into the ‘healthy immigrant effect’: health status and health service use of immigrants to Canada. Social Sci Med 2004; 59: 1613–1627. DOI: 10.1016/j.socscimed.2004.02.004. 15279920

[39] Laroche M . Health status and health services utilization of Canada’s immigrant and non-immigrant populations. Can Public Policy 2000; 26: 51. DOI: 10.2307/3552256. 18271124

[40] Ahmed S Shommu NS Rumana N , et al. Barriers to access of primary healthcare by immigrant populations in Canada: a literature review. J Immigr Minor Health 2016; 18: 1522–1540. DOI: 10.1007/s10903-015-0276-z. 26364053

[41] Fortin M Hudon C Haggerty J , et al. Prevalence estimates of multimorbidity: a comparative study of two sources. BMC Health Serv Res 2010; 10. DOI: 10.1186/1472-6963-10-111. PMC2907759 20459621

[42] Fortin M Stewart M Poitras ME , et al. A systematic review of prevalence studies on multimorbidity: toward a more uniform methodology. Ann Fam Med 2012; 10: 142–151. DOI: 10.1370/afm.1337. 22412006 PMC3315131

